# Using machine learning tools for protein database biocuration assistance

**DOI:** 10.1038/s41598-018-28330-z

**Published:** 2018-07-05

**Authors:** Caroline König, Ilmira Shaim, Alfredo Vellido, Enrique Romero, René Alquézar, Jesús Giraldo

**Affiliations:** 1 IDEAI Research Center, Universitat Politècnica de Catalunya, UPC BarcelonaTech, 08034 Barcelona, Spain; 2 Centro de Investigación Biomédica en Red en Bioingeniería, Biomateriales y Nanomedicina (CIBER-BBN), 08193 Cerdanyola del Vallès, Spain; 3 Institut de Neurociències - Unitat de Bioestadìstica, Universitat Autònoma de Barcelona, 08193 Cerdanyola del Vallès, Spain; 4 Network Biomedical Research Center on Mental Health (CIBERSAM), Madrid, 28029 Spain

## Abstract

Biocuration in the omics sciences has become paramount, as research in these fields rapidly evolves towards increasingly data-dependent models. As a result, the management of web-accessible publicly-available databases becomes a central task in biological knowledge dissemination. One relevant challenge for biocurators is the unambiguous identification of biological entities. In this study, we illustrate the adequacy of machine learning methods as biocuration assistance tools using a publicly available protein database as an example. This database contains information on G Protein-Coupled Receptors (GPCRs), which are part of eukaryotic cell membranes and relevant in cell communication as well as major drug targets in pharmacology. These receptors are characterized according to subtype labels. Previous analysis of this database provided evidence that some of the receptor sequences could be affected by a case of *label noise*, as they appeared to be too consistently misclassified by machine learning methods. Here, we extend our analysis to recent and quite substantially modified new versions of the database and reveal their now extremely accurate labeling using several machine learning models and different transformations of the unaligned sequences. These findings support the adequacy of our proposed method to identify problematic labeling cases as a tool for database biocuration.

## Introduction

In a very short period of time, the life sciences have become data-centric endeavors^1^. At the avantgarde of this trend, the omics sciences benefit from simultaneous rapid advances in computational systems and data acquisition technologies and now face data management challenges that go well beyond wet lab practice^2^.

Biological data in the omics sciences is usually curated by specially assigned professional scientists in a process often known as biocuration. It has been described as “the activity of organizing, representing and making biological information accessible”^3^ to biologists. It is becoming a key task, given that expert-curated web-accessible databases are one of the main driving forces in current research in biology in general and bioinformatics in particular^4^. The responsibilities of curators may include data collection; consistency, incompleteness^5^ and accuracy control; annotation using widely accepted nomenclatures; or evaluation of computational analysis, amongst others. Biocuration requires broad expertise in the domain because of the vast amount of heterogeneous information available from literature, often lacking a unified and standardized approach for the representation and analysis of data. This often involves a previously unforeseen forefront role for text mining methods^6^. One of the challenges of biocuration is the unambiguous identification of biological entities from existing studies and literature. Data trustworthiness can only be ensured through costly data management^7^. This task, when understood as “manual” expert curation, is uncertain and error-prone due to the complexity of the information involved, so that the development of computational procedures to assist experts in it is worth pursuing.

In this paper, we illustrate this using a specific example of how machine learning methods can be used to assist the curation of a protein database. This example involves a web-accessible and publicly-available database of G protein-coupled receptors (GPCRs). These are membrane proteins responsible for numerous physiological responses by transducing the signals embodied in the chemical structure of hormones, neurotransmitters and synthetic ligands and also the energy encapsulated in light photons from outside to inside the cells^8^. GPCRs are folded into seven helices that cross the cellular membrane and are connected by three intra- and three extracellular loops. GPCRs have a key role in regulating the central nervous system (CNS) function. Thus, it is not surprising that they have been among the most successful targets for the treatment of CNS disorders^9,10^. More than 800 human GPCRs exist, which constitute about 12% of human protein drug targets, and are, in turn, targeted by about 33% of currently marketed drugs, which makes them one of the most important target families in drug discovery programs^10^.

The first study that aimed to represent the overall map of the GPCRs in a single mammalian genome classified the human GPCRs in five main families or classes by phylogenetic analysis. These classes were termed glutamate, rhodopsin, adhesion, frizzled/taste2, and secretin (hence the GRAFS classification system)^11^. The glutamate family (also known as class C GPCRs), which is the subject of the present study, included the following receptor subtypes: eight metabotropic glutamate receptors, a GABA_*B*_ receptor heteromer composed of two subunits, a single calcium-sensing receptor, and five receptors that were believed to be taste receptors^11^. In a subsequent study, which was focused in class C GPCRs and performed in human (22 sequences), mouse (79), Fugu (30), and zebrafish (32) genomes, as well as in four invertebrate species, four main phylogenetic groups divided in eight subgroups were found^12^. Namely, Group I: V2R (pheromone receptor), TAS1R (sweet taste receptor), GPRC6A, and CASR (calcium-sensing receptor); Group II: GRM (mGlu receptors), Group III: GABA_*B*_ together with GPR158 and GPR158L and Group IV: GPRC5^12^.

From a structural point of view, class C GPCRs are characterized, in addition to the seven-helix transmembrane (7TM) domain, which is typical of all GPCRs, by a large extracellular domain (Venus flytrap or VFT) that in most cases is connected to the 7TM by a cysteine rich domain (CRD)^13^. In contrast with rhodopsin GPCRs (also known as class A GPCRs), which bind their endogenous ligands within the 7TM domain, most class C GPCRs bind their respective endogenous ligand within the VFT domain, thereby leaving the 7TM domain suitable for allosterism-based drug discovery^14–16^. The VFT is found only in Group I, II, and part of the group III (GABA_*B*_ subunits only). The absence of the VFT in some of Class C receptors has raised the hypotheses that either there is an endogenous ligand binding site at the 7TM domain for these receptors or they lack a ligand binding site and their function is related with allosteric effects through their potential heteromerization with other receptors^12^. Also the CRD is missing in Group III and IV Class C receptors. In the case of heterodimeric GABA_*B*_, their two subunits (GABA_*B*1_ and GABA_*B*2_) have different functional roles. Whereas the VFT of GABA_*B*1_ is responsible for neurotransmitter binding, the TM domain of GABA _*B*2_ is responsible for G protein binding^17^. As examples of the relevance of class C GPCRs as drug targets, metabotropic glutamate and GABA_*B*_ receptors are involved in various neurologic and psychiatric disorders amongst them Parkinson’s disease, schizophrenia and depression^18,19^.

Pharmacological databases are fundamental for the analysis of the structure and function of biological signal transduction entities, that is, receptors and ion channels^20^. GPCRdb^21^ is a web-accessible and publicly-available repository and information system containing data and web tools especially designed for GPCR research. Established back in 1993, it includes published information about the five major GPCR classes^11^. Class C, investigated in the current study, in turn comprises several subtypes. From GPCRdb, a class C dataset from March 2011 was object of extensive analysis using machine learning methods in our previous research^22–26^. These analyses revealed a possible receptor *label noise* problem^27^. Here, label noise implies the possibility that the sequence subtype labels, taken to be the ground truth, were wrong due to the uncertainty of the own database sequence labeling procedure. The problem takes the form of primary sequences being clearly and consistently misclassified by the machine learning methods as belonging to a different subtype than that reflected by their database label. The obtained results were understood as the first foundations for the development of a tool to assist omics database experts in their curator tasks by shortlisting items (proteins, genes) with questionable labels.

In the current study, we go one step further and track the evolution of the class C GPCR dataset in GPCRdb, which is a regularly updated database, by comparing the 2011 dataset with two recent and successive versions from 2016 (May and September). We compare the datasets regarding the number of sequences and the number of subtypes of the class C GPCRs as an assessment of the internal data quality of the datasets using machine learning techniques. More specifically, we use supervised classification methods and a detailed analysis of frequently misclassified items^24^.

In short, the possibilities of machine learning methods as database curation assistance tools are illustrated in this paper by using them to track the evolution of the GPCRdb database from 2011 to 2016 using the class C primary sequence data in order to find out whether the label noise problem might have been successfully tackled, ameliorating classification.

## Data

The GPCRdb^21,28^ is a curated and publicly accessible repository of GPCR databases and web tools for the analysis of membrane proteins including about 400 human specimens. Overall, the GPCRdb dataset contains 14,951 proteins from 3,184 species.

This resource has been available from 1993^29^ and its management was transferred in 2013 to the Department of Drug Design and Pharmacology at the University of Copenhagen in Denmark. The categorization of the receptors available form this database follows the international IUPHAR system recommendations. The whole database originally consisted of seven families: A (Rhodopsin), B1 (Secretin), B2 (Adhesion), C (Glutamate), F (Frizzled), Taste 2 and “other” GPCRs.

### Evolution of the database

As mentioned in the introduction, the computational experiments reported in this paper concern GPCRs of class C. At the highest level of grouping, class C discriminates receptors as *ion*, *amino acid*, or *sensory* according to the type of ligand. This study covers the evolution of GPCRdb over three versions: the first one released in 2011 and two recent and drastically changed versions: those of May 2016 and September 2016. At the second level of classification of the current database version, four subtypes are distinguished: metabotropic glutamate receptors (mG, amino acid), GABA_*B*_ (GB, amino acid), calcium sensing (CS, ion) and taste 1 receptors (Ta, sensory), covering sweet and umami tastes. The earlier 2011 version of the database also included three more sensory-related subtypes of the second level, namely vomeronasal (VN), pheromones (Ph) and odorant (Od) receptors.

**Figure 1:**
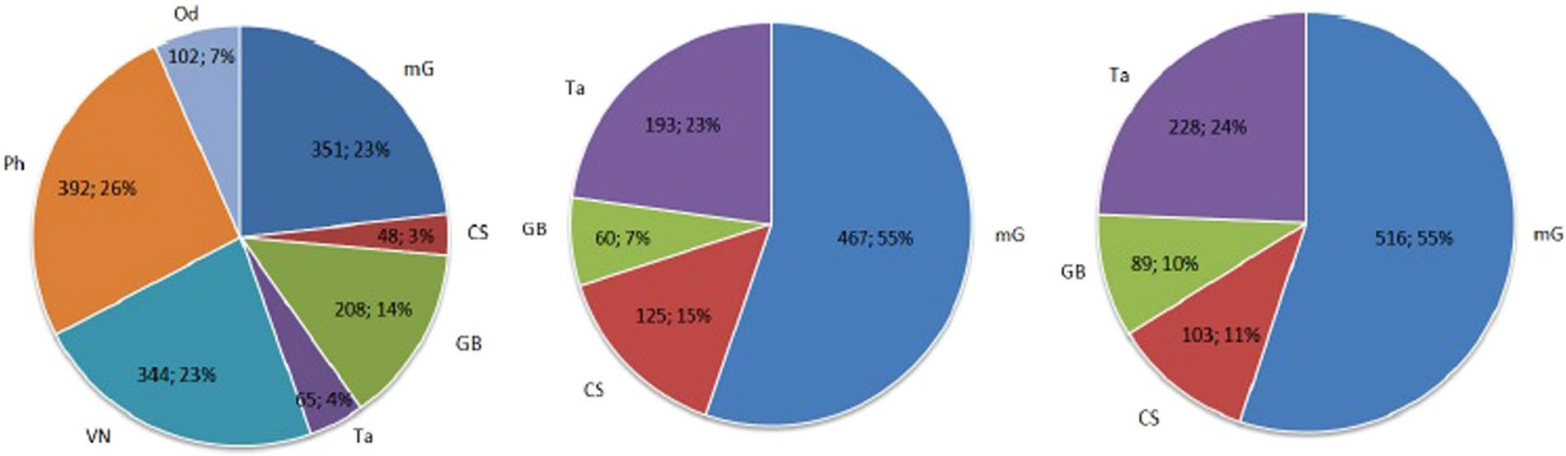
Subtype distribution (number of sequences and percentage) for the different databases: Left - March 2011, Middle - May 2016, Right - September 2016. Orphans are not included.

Over the five years elapsed between the earlier and later versions of the database analyzed in this study, GPCRdb has undergone major changes in the total numbers of proteins belonging to class C, but also in the ratio of the different subtypes to the total number of receptors and even in the sequences contained in each of those subtypes (see Table 1 and Fig. 1 for some summary figures).

**Table 1:** Number of receptors in each subtype for the class C GPCR datasets from the different database versions, including percentages of sequences preserved from one version to the next.

Subtype	2011	May 2016	Sept 2016	2011 ∩ May 2016	May 2016 ∩ Sept 2016
mG	351	467	516	93 (26%)	357 (76%)
CS	48	125	103	10 (21%)	91 (73%)
GB	208	60	89	10 (5%)	50 (83%)
Ta	65	193	228	42 (65%)	129 (67%)
VN	344	0	0		
Ph	392	0	0		
Od	102	0	0		
Orphans	147	193	18	0	18 (9%)
Total	1657	1038	954	155	645

Receptor acronyms as described in the main text. The last two columns reflect the intersection between different database versions.

The main changes occurred in the transition from the 2011 to the May 2016 versions, with only 155 protein sequences remaining unchanged. Not only the receptors of three subtypes (VN, Od and Ph) were removed in full from class C, but the number of proteins in the other remaining subtypes also changed significantly.

The mG receptors subtype grew by 33% and only 26% of sequences were kept unchanged (“2011 ∩ May 2016” column in Table 1). The CS receptors subtype more than doubled, keeping only 10 sequences unchanged. Finally, the Taste 1 subtype grew threefold (note that in the the 2011 version it was characterized simply as Taste), while the GB receptors subtype, on the contrary, decreased more than threefold.

The changes between the two 2016 versions are not so substantial, but still significant for a mere four-month period. In this case, the number of sequences kept completely unchanged varied from 65% to 85% for the four subtypes. The mG subclass kept growing in the September 2016 version by 10%; the GB and Taste 1 also increased by 50% and 18%, respectively. Instead, CS decreased by 18%. The largest of differences, though, was to be found in the number of orphan receptors (those not assigned to a subclass). Less than 10% of the original orphans were kept in the last version.

Note that only limited information regarding these sometimes drastic database changes is publicly available. In fact, little detail is known regarding the rationale behind those changes.

### Previous research on GPCR class C from a data curation perspective

Subtype classification of GPCRs has been attempted at different levels of detail^30^. Our interest in the analysis of the evolution of this database from a data curation perspective stems from early experiments^23^ in which we tested the extent to which class C GPCR first-level subtypes could be automatically discriminated from different transformations of their unaligned primary sequences.

Work on the 2011 version of the database provided evidence of clearly defined limits to the separability of the different class C subtypes. This evidence was produced using both supervised^25,26^ and semi-supervised^22^ machine learning approaches and from different data transformation strategies. Interestingly, the subtypes shown to be most responsible for such lack of complete subtype separability were precisely those which were removed in the 2016 versions of the databases (namely vomeronasal, odorant and pheromone receptors).

These results were further confirmed from the viewpoint of visualization-oriented fully unsupervised machine learning methods (that is, methods that attempted sequence discrimination into subtypes without knowledge of sequence-to-subtype assignment). Results clearly indicated that the subtypes shown to be worse discriminated by supervised classifiers were also those shown to heavily overlap in unsupervised visualization models from different unaligned sequence data transformations^31^.

These results might be just considered as a typical case of heterogeneous levels of subtype separability, often observed in real biological datasets. Closer inspection of the sequence misclassification behavior, though, revealed an intriguing and potentially more interesting pattern: different runs of the same, or even of different, classification algorithms, might be expected to yield different subtype predictions for the same sequences. That is, we might expect a given sequence to be misclassified only in part of the experiments and/or be misclassified to different subtypes. For instance, a receptor sequence might be misclassified in only a percentage of experiments, being perhaps sometimes predicted to be a CS receptor, while others predicted to be a GB receptor. Some of the observed misclassifications conformed to this typical pattern, but many others were found to be far too consistent, in the sense that the sequence was almost always misclassified (by different classifiers and different implementations of the same classifier) as belonging to the same *wrong* subtype.

This behaviour suggested that we might be witnessing a case of the *label noise* problem^27^. This is, the possibility that the sequence subtype labels as appearing in the database, taken to be the ground truth, were actually wrong as the result of the uncertainty of the own database sequence labeling procedure, very often model-based itself. This would explain both the presence of consistently misclassified proteins and (at least partially) the limits of subtype discrimination accuracy which our experiments stubbornly showed to exist, independently of the choice of data transformation and classification technique.

This problem was analyzed in detail in^24^, where individual sequences were identified and shortlisted as potential cases of label noise to be further analyzed by database curators. Unsurprisingly perhaps, most of them belonged to the same three subtypes previously identified as the most difficult to discriminate, namely VN, Ph and Od. All data transformations used in these experiments were alignment-free and included *n*-gram frequencies for *n* = 1, 2, auto-cross-covariance (ACC^32^) and the physicochemical distance transformation (PDT^33^). The classifier of choice was a Support Vector Machine (SVM), a model that has been widely favoured for this type of problems (see, for instance^33–35^).

Subsequent work reported in^26^, which again employed alignment-free data transformations, used a Random Forest (RF) classifier^36^ to further investigate the consistency of misclassification in this problem. Note that RF is an ensemble learning technique^37^ with an internal classification *voting* system that is naturally suited to classification consistency analyses. The classification performance achieved with RF was similar to that of SVM across transformations. Most consistent misclassifications were again detected mainly in VN, Ph and Od, confirming our previous results.

All these studies were based on the earlier 2011 version of the database, which automatically raised the following research question: if the 2011 database, which included VN, Ph and Od as Class C GPCR subtypes, suffered from these label noise classification problems, would the new 2016 versions of the database, which do not include those subtypes, suffer from similar problems? This is the question we aim to answer through the experiments reported next.

## Results

In this Section we detail the experimental results of the analyses of the three different datasets. We report the classification results obtained using different supervised classifiers for the transformed primary sequences of the proteins applying 5-fold cross validation (CV). Tables 2 and 3 show, in turn, the classification results for the datasets published on March 2011, May 2016 and September 2016. In each table, several evaluation measures are reported for SVM, Naive Bayes (NB) and RF classifiers, as well as for five different transformations of the primary sequences (described in detail in the Methods section): the Amino Acid Composition (AAC), Digram Composition (Digram), Auto-cross covariance (ACC) and two variants of Prot2Vec: the first based on a Swiss-Prot database representation and the second based on a GPCRdb representation. Details about the classifiers and the data transformation methods are provided in the Methods section.

**Table 2:** Classification results for the 2011 version dataset. *Prot2Vec1* corresponds to the Swiss-Prot-based representation and *Prot2Vec2* corresponds to the GPCRdb-based representation.

Model	Classifier	Accuracy	MCC	F-measure
AAC	SVM	0.8855	0.8549	0.8842
	RF	0.8570	0.8207	0.8542
	NB	0.7033	0.6307	0.7046
Digram	**SVM**	**0.9311**	**0.9128**	**0.9303**
	RF	0.9139	0.8929	0.9124
	NB	0.8358	0.7949	0.8375
ACC	SVM	0.9252	0.9054	0.9234
	RF	0.8894	0.8624	0.8838
	NB	0.8430	0.8064	0.8455
Prot2Vec1	SVM	0.8987	0.8715	0.8981
	RF	0.8596	0.8245	0.8587
	NB	0.6000	0.5153	0.6070
Prot2Vec2	SVM	0.8695	0.8353	0.8692
	RF	0.8093	0.7625	0.8110
	NB	0.5854	0.4931	0.5889

**Table 3:** Classification results for the May and September 2016 version datasets respectively.

		May 2016			Sept. 2016		
Model	Classifier	Accuracy	MCC	F-measure	Accuracy	MCC	F-measure
AAC	SVM	0.9822	0.9714	0.982	0.9893	0.9824	0.9892
	RF	0.9716	0.9538	0.9706	0.9850	0.9757	0.9850
	NB	0.9550	0.9271	0.9551	0.9594	0.9368	0.9598
Digram	SVM	0.9917	0.9884	0.9916	0.9946	0.9925	0.9946
	RF	0.9905	0.9847	0.9905	0.9914	0.9860	0.9914
	NB	0.9811	0.9688	0.9808	0.9893	0.9826	0.9893
ACC	**SVM**	**0.9941**	**0.9917**	**0.994**	**0.9968**	**0.9951**	**0.9968**
	RF	0.9893	0.9830	0.9891	0.9925	0.9878	0.9925
	NB	0.9799	0.9673	0.9798	0.9904	0.9845	0.9903
Prot2Vec1	SVM	0.9822	0.9716	0.9822	0.9893	0.9839	0.9893
	RF	0.9763	0.9612	0.9759	0.9861	0.9776	0.9861
	NB	0.8118	0.7229	0.8207	0.9904	0.9845	0.9903
Prot2Vec2	SVM	0.9822	0.9759	0.9823	0.9936	0.9912	0.9936
	RF	0.9822	0.9714	0.9821	0.9904	0.9847	0.9903
	NB	0.8615	0.7972	0.8688	0.9808	0.9692	0.9809

The best classification results were obtained with the SVM classifier for all three datasets and across all transformations (with minor exceptions for *prot2Vec1* in the September 2016 dataset). Tables 4 and 5 detail the SVM classification results for the best performing transformations at the subtype level.

**Table 4:** Subtype classification results obtained by SVM from the Digram transformation of the 2011 version dataset.

Subtype	Precision	Recall	MCC	F-measure
mG	0.9462	0.9829	0.9639	0.9532
CS	1.0	0.9356	0.9645	0.9652
GB	0.9905	0.9856	0.9880	0.9861
Vn	0.9185	0.9128	0.9153	0.8907
Ph	0.8980	0.9131	0.9050	0.8719
Od	0.8610	0.7362	0.7896	0.7806
Ta	1.0	0.9846	0.9920	0.9918

**Table 5:** Subtype classification results obtained by SVM from the ACC transformation of the May and Sept. 2016 version dataset respectively.

Subtype	May 2016				Sept. 2016			
	Precision	Recall	MCC	F-measure	Precision	Recall	MCC	F-measure
mG	0.9958	1.0	0.9979	0.9953	0.9962	1.0	0.9981	0.9957
CS	0.9923	0.9760	0.9833	0.9811	1.0	0.9804	0.9899	0.9889
GB	1.0	0.9833	0.9913	0.9909	1.0	0.9889	0.9943	0.9938
Ta	0.9903	0.9949	0.9924	0.9902	0.9957	1.0	0.9979	0.9972

A detailed analysis of the consistently misclassified sequences reveals no coincidence with the results from the study of the 2011 database^24^, for the obvious reason that none of the 11 sequences reported as consistently misclassified in this study is part of the 2016 databases (for a formal description of the misclassification consistency concept, we refer the readers to the Methods section). A study of the misclassifications of the 2016 database reveals that only the sequence *h*2*u*5*u*4_*takru*, labeled as GB, is misclassified for all 5 data transformations of the present study. Nevertheless the prediction of class membership of this sequence is not completely uniform, as it is predicted to belong to Ta in 4 cases and to mG in one case. This is, according to Uniprot, an uncharacterized protein, i.e. inferred from homology. Sequence *t*2*mdm*0_*hydvu* was also detected as frequently misclassified (for 4 out of 5 transformations). This sequence is labeled as mG, but the classifiers predict it to belong to CS. Table 6 details the measures employed to analyze the consistency of misclassification of these two sequences.

**Table 6:** Analysis of misclassification of sequences *h*2*u*5*u*4_*takru* and *t*2*mdm*0_*hydvu*: For each sequence *s* the true class (TC), the predicted class (PC), the *error rate* (*ER*_*s*_), the *voting ratio* (*R*_*s*_) and the *cumulative decision value* (*CDV*_*s*_) are reported. For the meaning of these measures, see the *Methods* section.

	h2u5u4_takru					t2mdm0_hydvu				
Model	TC	PC	*ERs*	*Rs*	*CDVs*	TC	PC	*ERs*	*Rs*	*CDVs*
AAC	GB	Ta	100	0.49	38.18	mG	Ta	100	0.34	−59.58
Digram	GB	Ta	96	0.51	−9	mG	Ta	100	0.38	28.75
ACC	GB	mG	100	0.46	19.16	mG	mG	0	—	—
Prot2Vec1	GB	Ta	100	0.58	−42.54	mG	CS	100	0.33	55.5
Prot2Vec2	GB	Ta	100	0.41	−28.52	mG	CS	100	0.39	−10.36

## Discussion

Note that the main goal of this study is to illustrate the use of machine learning methods as protein database curation assistance tools. The case study focuses on the comparative analysis of class C GPCR data over time using three versions of a publicly available database spanning from 2011 to 2016. This analysis concerns the ability of different machine learning methods to discriminate between class C subtypes from different transformations of their unaligned sequences. Such discriminability analysis is geared towards the assessment of the *label noise* problem observed in our previous investigation of the 2011 version datasets and is meant as a way to assist database experts in their biocuration tasks.

The mere comparison of the datasets shows a remarkable reduction of the number of sequences, from the 1,510 sequences in the March 2011 dataset, down to the 936 collected in the September 2016 one, not counting orphans. Moreover and as previously mentioned, the variety of subtypes included in class C has been reduced from the seven of the 2011 dataset to only four in both 2016 datasets.

The results of the analyses of the datasets using supervised classification methods, reported in the previous section, lead to some unequivocal conclusions.

According to the results in Tables 2 and 3, all classifiers perform better with the 2016 datasets than with the 2011 dataset according to all the evaluation measures considered. Furthermore, the September 2016 version of the dataset yields consistently better results that the May 2016 version although, in this case, differences are comparatively minor.

It might be argued that the differences between the 2011 and 2016 datasets could be put down to the fact that the VN, Ph and Od subtypes have been removed from the 2016 versions. This is true only to some extent because, importantly, the subtype-specific results in Tables 4 and 5 indicate that the 2016 versions yield better performance than the 2011 version for each and every of the four remaining subtypes independently (remarkably for mG and CS). And again, the September 2016 results are slightly better than the May 2016 results for each of the four subtypes.

An accuracy of 0.9941 using the SVM with ACC transformation for the 845 sequences of the May 2016 version dataset implies just 6 misclassifications. Correspondingly, a 0.9968 accuracy, also for the SVM with ACC for the 936 sequences of the September 2016 version dataset, implies 3 misclassifications. These are almost negligible numbers when compared to those of the 2011 version. Moreover, note that out of these few cases and as reported in the previous section, only a couple of sequences show the type of very consistent misclassification that might be evidence of label noise: *h*2*u*5*u*4_*takru*, labeled as GB and predicted to probably belong to Ta and *t*2*mdm*0_*hydvu*, labeled as MG and predicted to belong to CS or Ta. In comparison, the results from the study of the 2011 database^24^, using the same criteria as the current study, indicated the existence of a shortlist of at least 11 very consistently misclassified sequences even when an extremely conservative threshold was used to assess such consistency. In our opinion, this is evidence of sound curation at work, as well as evidence of how important it is to use label noise detailed assessment as a tool to assist biocuration.

We can also conclude that SVM classifiers show a very consistent overall advantage when compared to RF and NB for all three datasets and for all five data transformations. The difference is very clear with the 2011 version and more nuanced with the 2016 datasets. This is a relevant result for two reasons: first, because it reveals SVM performance to be more robust in datasets with limited class separability; second, because it reveals that with neatly separable classes such as those of the 2016 datasets, almost any classifier will do reasonably well, even the baseline NB classifier. This is further evidence that sound biocuration, when dealing with the label noise problem adequately, helps to reduce the uncertainty associated to model-based decision making, in this case by limiting the impact of the choice of data analysis methods (here, the choice of classifiers) on the results.

Finally, we should consider the impact of the data transformations on the classification results. The interpretation of the corresponding comparative results bears similarities with that of the comparative of classification methods. Digram performs best for the 2011 version of the database, while the more complex ACC performs best for both 2016 versions. Again, the differences in performance between transformations for all classifiers are relatively small for the 2016 datasets and no transformation with no classifier falls below the 0.98 accuracy mark with the September 2016 dataset. Therefore, this again reinforces the idea that biocuration, by dealing with label noise, reduces the uncertainty associated to model-based decision making, in this case by limiting the impact of the choice of data transformation method on the results. A last comment on this issue is that the recently proposed (and most complex of our choices in this study) Prot2Vec transformation^38^ does not seem to show any relative advantage for the analyzed data.

Our experiments quite conclusively indicate that the last 2016 version of the class C data in GPCRdb, a reference for GPCR research, is almost free of the *label noise* problem. That is, almost none of the class C GPCR sequences in this version is predicted by our machine learning-based method to be consistently misclassified. In other words, the method considers that, even if misclassifications still exist, almost none of them should be suspected to be a labelling error. Having tracked this database from 2011 according to this criterion, we are now in a position to confidently say that the analysis of label noise in this type of databases, understood as a problem of misclassification consistency, is a useful tool for biological database curation.

Importantly, and despite the fact that the research reported in this paper has focused on class C GPCRs as a case study, the proposed method could be *exported* to any omics database in which biological entities are associated to a characterization label. This research also highlights the importance of documenting the reasons for changes between versions of publicly available biological databases.

## Methods

Our experiments involve the supervised classification of transformed versions of unaligned^39^ primary amino acid (AA) sequences of class C GPCRs. Transformations are required to achieve fixed length sequence representations.

The transformed datasets were analyzed with SVMs^40^, but also with NB^41^ and RF^36^ classifiers for comparison. All these classifiers are now standard in bioinformatics research and are different enough as to provide a well-informed comparative of results.

### Data Transformations

Several AA sequence transformation approaches were considered: First, we used *n*-gram based transformations that treat symbolic sequences as *text* from a 20 AA alphabet^42^. This transformation was used in^25^ for the 2011 dataset. Second, we used transformations based on the physicochemcial properties of the AA, which were reported as novel methods in^43^ and^44^ and were used for the 2011 dataset in^22–24^. The third type of transformation are based on continuous skip-gram models.

- The Amino Acid Composition (AAC) transformation measures the frequency of appearance of each of the 20 AA in the sequence, yielding vectors of length 20.
- The Digram Composition (Digram) measures the frequency of appearance of each *n*-gram of length 2 in the primary sequence (20 × 20 possible combinations), yielding vectors of length 400.
- The Auto-Cross Covariance (ACC) transformation^32,45^ transforms the primary sequence according to the AA physicochemical properties. First the AA sequence is transformed to a 5-dimensional vector of *z*-scores^46^ for each AA, representing its physicochemical properties. The Auto Covariance (AC) and Cross Covariance (CC) of these *z*-scores are then computed for residues separated by a maximum lag *L*. They measure, in turn, the correlation between the same descriptors or different descriptors. The resulting vector is the concatenation of all AC and CC terms from lag *l* = 1 up to *L*. For each dataset we estimated the lag *l* that yields the transformation with best classification results. For the 2011 dataset, ACC was calculated for *l* = 13 resulting in vectors of size 325, which provided the best classification results in^22^. The same parameters were used for the 2016 datasets.
- *Prot2Vec* distributed transformations: This is a natural language processing (NLP)-inspired transformation. To apply it to protein sequence classification, the AAs are considered as letters and the whole sequences as sentences, with *n*-grams acting as words. In NLP, this representation is understood as “distributed” because one “concept” in the domain is represented in several dimensions and one dimension gathers information about several “concepts”. In NLP, these distributed word representations are learnt using an Artificial Neural Network model and have been refined in the form of Continuous Bag-of-Words (CBOW) and Continuous Skip-Gram (CSG) models^47^.

This idea was extended to protein sequences in^38^, where it was shown to capture meaningful physical and chemical properties of the proteins. In the current work, 3-gram representations were first created from two different databases: Swiss-Prot and GPCRdb. The GPCRdb representation was created using the complete database (not only class C) for the May and September 2016 versions. To train the model, each sequence was split into 3 sequences of 3-grams with offsets from 0 to 2 that were used in training set. A skip-gram version of window size 25 was used to train both models. For the final working represention of a sequence, the vectors corresponding to its 3-grams were summed up.

### Supervised classification

The subtype discrimination problem is addressed as multi-class classification, where a class C GPCR subtype label is predicted from the transformed unaligned primary sequences. The 2011 version dataset comprises seven subtypes, while the 2016 datasets comprise four subtypes. As previously mentioned, the experiments involve SVM, NB and RF classifiers.

#### Support Vector Machines

SVMs have become a standard method of choice for protein classification problems, in variants such as SVM-HUSTLE^48^, SVM-I-sites^49^, SVM-n-peptide^50^ and SVM-BALSA^51^, amongst others. They are based on statistical learning theory^40^ and aim to separate the data items according to class label with a maximal margin, while minimizing the classification error *ξ*. The use of non-linear kernel functions allows SVMs to separate input data in higher dimensional spaces, which would not be separable with lower complex linear classifiers. A common choice is the radial basis function (RBF) kernel, specified as $K({x}_{i},{x}_{j})={e}^{(-\gamma ||{x}_{i}-{x}_{j}||)}$. Using a RBF kernel the SVM needs to adjust two parameters, the error penalty parameter *C* and the *γ* coefficient, through grid search. Our problem involves multi-class classification for which a “one-against-one” approach is used and implemented in the LIBSVM library^52^.

#### Naive Bayes

NB^41^ is a simpler model that provides a baseline for comparison. It is a probabilistic classifier which applies Bayes’ theorem with an assumption of independence of variables. Under this assumption the probability of a class given the input data is expressed as $P({C}_{i}|X)=P({C}_{i}){\prod }_{n=1}^{N}P({X}_{n}|{C}_{i})$. This probability could be used for class prediction using Maximum A Posteriori (MAP) estimation in the form $y=arg{max}_{i}P({C}_{i}){\prod }_{n=1}^{N}P({X}_{n}|{C}_{i})$. The classifiers differ depending on the assumption about the probability distribution for *P*(*X*_*n*_|*C*_*i*_). For continuous variables the typical assumption is a Gaussian:

$$P({X}_{n}|{C}_{i})=\frac{1}{\sqrt{2\pi {\sigma }_{n}^{2}}}{\exp }^{-\frac{{X}_{n}-{\mu }_{n}}{2{\sigma }_{n}^{2}}}$$

The parameters *μ*_*n*_ and *σ*_*n*_ are estimated using Maximum Likelihood.

#### Random Forest

RF^36^ is an *ensemble learning* method^37^ using decision tree (DT)-based classifiers. The DT classifiers are trained to split an input space into homogeneous regions with associated class labels. The splits are typically axis-aligned and are selected to maximize the *information gain*.

### Classification performance metrics

Several metrics were used for the evaluation of the classifiers’ performance. At the subtype level, i.e. for the binary classifiers of each subtype, the performance was evaluated using the Precision (as measure of quality), the Recall (as measure of completeness), the Matthew Correlation Coefficient (MCC) and the F-measure. The latter two are considered more robust evaluation measures as MCC takes into account all elements of the confusion matrix making it suitable for unbalanced datasets^53^, while the F-measure involves only Precision and Recall. All these metrics are based on the concept of true and false predictions in binary classification according to^54^. The MCC, defined as correlation coefficient between the observed and the predicted classification, ranges from −1 to 1, where 1 corresponds to a perfect classification, 0 to a random classification and −1 to complete misclassification. The F-measure being the harmonic mean of Precision and Recall ranges from 0 (describing complete misclassification) to 1 (perfect classification). For the multi-class classifiers, at the global level, we report the accuracy (Accu), which is the ratio of correctly classified sequence to their total number, but also the MCC and the F-measure as explained in^54,55^. The reported measures are the mean values of the respective metrics over the five iterations of the 5-fold CV used to evaluate the classifiers.

For the multi-class classifiers, at the global level, we report the accuracy (Accu), which is the ratio of correctly classified sequence to their total number, but also the MCC and the F-measure as explained in^54,55^. The reported measures are the mean values of the respective metrics over the five iterations of the 5-fold CV used to evaluate the classifiers.

### Systematic analysis of classification errors

We use a systematic three step approach to analyze the SVM classifier models built on the transformed dataset in order to assess the kind of classification error:

1. Using repeated CV, as proposed in^56^ and applied in^57^, the frequency of misclassification in the test set or *error rate*, *ER*_*s*_, is found for each sequence *s*. To this end, 100 iterations of resampled 5-fold CV were applied for classification. Sequences *s* misclassified with *ER*_*s*_ ≥ 75% are selected for further analysis.
2. For each sequence *s* detected as consistently misclassified in *step 1*, the *voting ratio R*_*s*_ = *VT*_*s*_/*VP*_*s*_ is evaluated, where *VT*_*s*_ and *VP*_*s*_ are the total number of votes obtained by, in turn, the *true* class and the most frequently predicted class in all the binary SVM classifiers in the 100 iterations. *R*_*s*_ ≤ *θ*_*R*_ = 0.5 is taken to indicate a consistent classification error.
3. For each sequence *s* selected in *step 1*, we evaluate the confidence of the SVM binary classifiers between its *true* class *i* and its most-predicted class *j* by defining a *cumulative decision value*, *CDV*_*s*_, as the sum of *DV*_*s*_(*i*, *j*, *k*), i.e. the decision value given by the binary SVM classifier confronting classes *i* and *j* for sequence *s* and *k* = 1, .., 100 test iterations. The magnitude of the error is deemed *large* if *CDV*_*s*_ ≥ *θ*_*CDV*_ = 60 in absolute value. The information conveyed by *CDV*_*s*_ complements that of *R*_*s*_, as explained in^24^.

### Data availability statement

The GPCR datasets analyzed in this study are publicly available from GPCRdb (http://gpcrdb.org). The remaining generated datasets are available upon request to the corresponding author.
